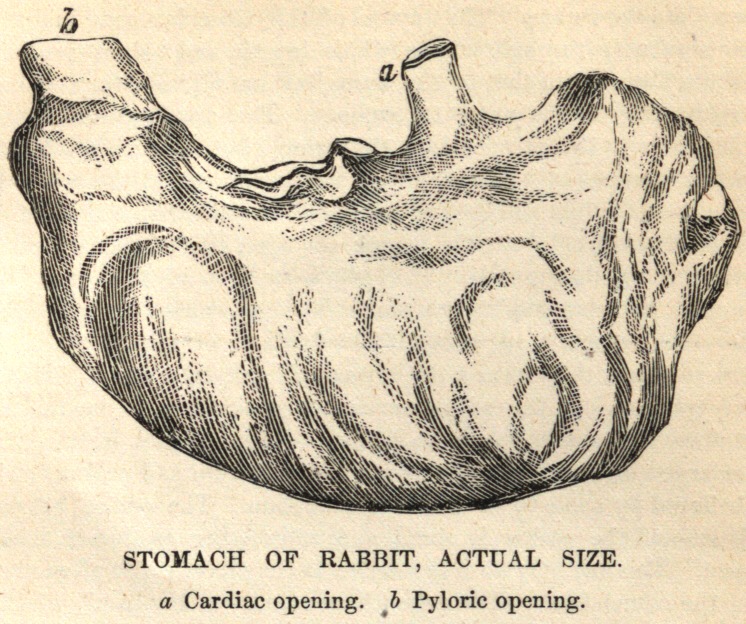# The Physiology of Digestion in Ruminants, with Practical Remarks*From notes of lectures on Physiology delivered by Prof. C. L. Dana, M.D., before the Columbia Veterinary College and School of Comparative Medicine.

**Published:** 1882-04

**Authors:** Henry C. Slee


### ART. XI.—THE PHYSIOLOGY OF DIGESTION IN RUMINANTS, WITH PRACTICAL REMARKS.*

BY HENRY C. SLEE.

The proverbial adaptability of nature to existing needs and circumstances is most beautifully illustrated in the digestive organs of ruminants. These creatures are naturally handicapped in the struggle for existence. Their food contains but a small amount of nourishment in a large matrix of waste material, requiring an immense amount of preparation before the nutritious portions can be absorbed. But by the wonderful formation of their digestive organs, they overcome this difficulty, and thrive and fatten where the unfortunate possessors of less complex organs could hardly subsist; hence we find them existing in all parts of the world, except New Holland and some of the South Sea Islands—braving alike the snows of Greenland and the lightning of the Alps; the thirsty deserts of the East and the dizzy peaks of the Cordilleras—and we find them in countless numbers on the luxuriant plains of Africa and America.

The substances from which this digestive machine can extract nutrition are astonishing. The reindeer grows fat on the moss which he finds underneath the snow, and for which he digs with his antlers—naturally he never eats grass or hay; the elk lives principally upon heather, reeds and twigs, and will nip off clean, with his lips, and swallow and *digest*, a twig that would almost serve for a riding whip; the goat will luxuriate in a diet of poisonous hemlock and aconite; and the average diet of the camel is so dry and hard that if he were provided with green spectacles and fed on shavings he would scarcely detect the fraud.

### THE ORGANS OF PREHENSION.

The organs of prehension vary somewhat in ruminants. In some, as in the cow, the lips, though strong, are not sufficiently movable to be of any use in prehension, while the tongue is so long and the muscles by which it is moved so powerful that it is the principal organ used in this process, like the lips in the horse. In the giraffe the tongue is generally about eighteen inches long. In some other rumi-

* From notes of lectures on Physiology delivered by Prof. C. L. Dana, M.D., before the Columbia Veterinary College and School of Comparative Medicine.

nants, as the sheep and goat, the tongue is even smaller proportionately than that of the horse, while the lips in these animals are very thin and movable, and are the principal organs of prehension. The upper lip of the sheep, camel, llama, deer and others is divided in front, enabling them to move either side independently of the other. The lips of the giraffe aud elk are very extensible, and those of the latter have fleshy appendages almost like a set of muscular teeth.

The interior of the cheeks of ruminants, are provided with large papillæ, which in the elk are of very large and in the camel have much the appearance and size of wooden shoepegs.

The teeth of ruminants are less in number than those of the horse, being generally thirty-two, and, with the exception of the camel and the llama, they have no upper incisors, these being replaced by a cartilaginous pad. The lower incisors are sometimes fixed in the jaw in such a manner as to admit of a slight amount of motion, and thus graduate the pressure upon the pad—a condition often mistaken by novices for disease. The molars are the same in number as those of the horse, but differently proportioned in size and the shape of their grinding surfaces. With the exception of the camel and the llama and a few species of deer, the ruminants have no canine teeth. The camel is the only one that possesses such teeth in the lower jaw. During fœtal life the ruminants often possess incisor teeth which never cut through.

THE SALIVARY GLANDS

are large in ruminants—the parotid is large in all; but the others vary in their relative proportions in different species. The sub-maxillary is the most prominent in the cow. These glands secrete a large amount of fluid, but it contains, as a rule, no diastase like the saliva of man, the horse, etc.

The camel is provided with what might almost be called an extra tongue, in the shape of a flap attached to the front of the soft palate, sometimes nearly a foot long, reaching down into the œsophagus. It can be protruded from the mouth like the tongue, with a peculiar noise. It secretes a watery fluid, catches water regurgitated from the reticulum, and moves over the tongue and pharynx, bathing them and relieving thirst.

A very prominent peculiarity of the digestive organs in ruminants is their proportional distribution. In them, the food is subjected to elaborate preparation and is pretty thoroughly digested before it reaches the small intestines, and the intestines are chiefly devoted to absorption; unlike the solipeds, in which there is little such preparation, and in which digestion is mainly carried on in the intestines. Hence we find in ruminants immense receptacles for the food, anterior to the intestines. While the horse's stomach will contain about fifteen quarts and his intestines two hundred quarts, the cow's "four stomachs" will contain nearly two hundred quarts and her intestines only eighty, viz.:

| | | |
|---|---|---|
| Rumen | 140 | quarts. |
| Reticulum | 4 | " |
| Psalter | 52 | " |
| Abomasus | 20 | " |
| Small intestine | 50 | " |
| Large intestine | 30 | " |

*The Rumen*, or paunch, is considerably larger than the other stomachs—that of the cow being large enough to contain over one hundred quarts; that of the goat, twenty to twenty-five quarts, and that of the sheep, twenty-five to thirty quarts—and occupies about three-quarters of the abdominal cavity, in which it is situated somewhat obliquely, touching the left abdominal wall at the flank. It is divided into two almost complete sacs by a strong muscular ridge, which commences at the left side of the cardiac orifice, and extends the full length of the sac. Each division is again subdivided. In the camel, this ridge sends out fibres at right angles, and these fibres are again connected by other sets of fibres, which form a series of cells for the retention of water. These are partially closed by a continuation of the lining membrane, leaving a small circular opening in the centre of each. In the full-grown dromedary the largest of these cells, when dilated, have a depth and width of three inches.

The mucous membrane lining the rumen has no mucous glands, but is covered by a particularly hard, firm pavement epitheleum, and in horned ruminants is covered with tufts, projecting from the surface, which vary in shape and size in different portions of the sac, being smallest near the muscular ridges. Their appearance is familiar in tripe. They vary in size in different species of ruminants, being larger in wild ruminants than in those which have been domesticated for ages, reaching the most extraordinary development in the gazelle, but being entirely absent in the camel. In the cow they vary in different portions of the rumen, some tapering almost to a point, and notched on the edges like an oak leaf. In the sheep and goat they are longer than in the cow, and their free margins are thin and spread out. In the bison they are large and coarse; in the reindeer and giraffe they grow larger as they rise from the surface; and in the reindeer they are interlaced. These variations are no doubt made to accord with the peculiar food of the animal.

The muscular walls of the paunch are composed of striated fibres, showing that their movements are under the control of the will—a remarkable deviation from the rule in animal creation—the muscles which control organic life being, in almost all other cases, non-striated.

The *second stomach*, or reticulum, is the smallest in the cow and in most other ruminants, and appears like a division of the rumen, from which it is separated by a thick muscular wall, in which is left a very large aperture. This aperture, is closed by a kind of valve, which allows the food to pass from the rumen into the recticulum, but prevents its return. The muscular walls of the reticulum are very powerful, and the mucous membrane lining it is folded in such a manner as to form narrow pits or crypts, giving it an appearance from which the names reticulum or honeycomb (by both of which it is known) are derived. The walls of these pits contain muscular fibres, so arranged that, by the contraction of one set, the pits will retain the water with which they become filled, and by the contraction of another set the pits are opened and their contents let out to moisten the food. This structure is most prominent in the camel, in which the reticulum is called the "water bag," and their subdivision into small cells is more minute than in the cells of the rumen. Their development varies in different species of ruminants, apparently in proportion to their need of water. In the cow, they are subdivided almost like those in the camel; but in the goat the subdivision is less marked. In the reindeer, which swallows a good deal of snow with its food, they are very shallow; and in the giraffe, which feeds principally on young leaves and buds, which contain a good deal of moisture, they are merely represented by raised lines—still, however, retaining the same uniform manner of division. In the musk deer—a rather curious, half-developed ruminant—the reticulum is barely recognizable, being scarcely divided from the rumen, and devoid of cells.

It is in the recticulum that the indigestible bodies so often swallowed by ruminants are generally lodged, and here and in the rumen the masses are formed which cause so much annoyance to stock-raisers. Similar formations are sometimes found in the intestines of the horse, and in both animals they are generally composed of a tribasic phosphate of ammonia and magnesia. Analysis of some has shown them to contain: phosphates, 80 per cent.; calcium, 5 per cent.; water, 15 per cent. In ruminants the nucleus of the mass is generally hair, with an accumulation of the above salts about it. These may be produced by feeding upon rye bran, which is rich in phosphates and magnesia. It is not very long since similar "stones" found in the intestines of the antelope, and known under the name of bezoar, were hawked through Europe as a general panacea for all diseases, increasing in value with their size, one weighing four ounces having brought the sum of $1,000.

The *third stomach*, which has been given many names, the most preferable of which seems the "psalter" (derived from the peculiar book-like folds of its lining membrane), is a wonderful piece of mechanism. These folds commence at the end where the œsophagus enters and run lengthwise the full length of the sac, the longest terminating at the aperture connecting this with the fourth stomach. On each side of every large leaf is another one-half as wide, and besides these, again, third leaves one-half the width of the last; and finally, between each set of folds, a distinct elevation of the membrane, as though still another fold had begun to be formed. The faces of these leaves are studded with small, hard nodules, thus forming a most perfect triturating apparatus, as there are muscular fibres running in the leaves by which they are moved and the food slowly rubbed together. These folds are generally fifteen in number, the largest of them wide enough in some ruminants to stretch half way across the sac. In the cow these folds are considerably developed, but in the camel they are merely rudimentary. The purpose of this we will see later on.

In the sheep and goat this stomach is smaller than the recticulum; in the camel it is very long; and in the musk deer it is scarcely divided from the fourth stomach, of which it seems a part.

STOMACH OF RABBIT, ACTUAL SIZE.

*a* Cardiac opening. *b* Pyloric opening.

The *fourth, or digesting* stomach, called the abomasus, is second in size to the paunch, and considerably more cylindrical in shape than that of the horse. It presents less variations than the other stomachs. In the cow and most other ruminants, its whole mucous lining is covered with glands which secrete gastric juice. In this respect it approaches more nearly that of the carnivora than does that of the horse, in whose stomach the gastric follicles cover not much more than half the surface. In the camel, however, this stomach is very small, compared with that of the cow, and the gastric surface is also less, being confined to the posterior two-thirds. The pyloric orifice is provided with a peculiar valve in the shape of a powerful muscle; but it is not tight, as in the horse.

We have left the consideration of the *œsophagus* until now, because, if not the most wonderful, it is the most complicated portion, and requires some knowledge of the stomachs to appreciate its mechanism. It is extremely dilatable throughout its whole extent, and is considerably wider, proportionally, than that of non-ruminating herbivora, more nearly resembling, in these respects, the œsophagus of the carnivora. It terminates in a funnel-shaped enlargement at its entrance into the rumen. The layers of the muscular coat maintain their regularity throughout the whole length, and do not terminate in white fibres, as in that of the horse, but are all voluntary; hence, its dilatability is the same throughout. The œsophagus does not terminate with its entrance into the rumen, but continues along the superior portion of the reticulum, and terminates at the orifice between the reticulum and the psalter. This continuation is in the form of a partly-inverted gutter or trough, the open side of which presents to the left and downwards. The edges, or lips, of this gutter are muscular, and rise gradually from their origin, where the œsophagus enters the rumen, until, at their termination, or entrance into the third stomach, they form a thick, powerful, almost perfect ring, but never quite a ring, the edges never being attached to one another, but always separable, except in infancy. This channel, in the camel, is enlarged into a small sac between the reticulum and psalter, which is believed by some to be the true reticulum. The orifice between this sac and the psalter is small, and, as in other ruminants, can be closed. This sac may have given rise to the assertion, so often made, that the camel has a fifth stomach or extra "water bag," which is now well known to be untrue.

In young calves, the first and second stomach are very small, being only gradually developed after weaning; and the orifices leading into them from the œsophagus are closed, the lips of this gutter adhering so as to form a tube leading directly into the third stomach; hence sucking calves do not ruminate.

The proportional capacity of the

INTESTINES OF RUMINANTS

and solipeds has been referred to. In ruminants they are small in calibre, though very long. The small intestine of a cow is $\frac{3}{4}$ to 1 inch in diameter; of a horse, 1 to $1\frac{1}{2}$ inches, and the colon of the cow is only $1\frac{1}{2}$ to $1\frac{3}{4}$ inches in diameter. The length is as follows:

| | Horse. | Cow. | Sheep. | Goat. |
|---|---|---|---|---|
| Average length of small intestine (feet) . . . . . . | 70 | 115 | 70 | 70 |
| Large intestine (feet). . . . | 16 | 35 | 20 | 25 |
| Proportion to length of body . . . . . . . . . . . . . | 1–12 | 1 to 18 or 20 | 1 to 26 or 28 | 1 to 26 or 28 |
| Superficial area (square yards). . . . . . . . . . . . | 15.5 | 15 | Pig 3 | Dog 5 |

The small intestine, at its commencement, is considerably enlarged in all ruminants; and in the camel this enlargement is very prominent, forming a sac half as large as the abomasum. In some fœtal ruminants the small intestine is of large calibre, bearing the same proportion as that of the horse.

The cæcum is smaller than in the horse and not puckered, except in the buffalo, in which it is bifid at the end.

The colon is not divisible into double and floating colons as in the horse, and is longest and smallest, and the coils most regular in those which expel the fæces in small pellets, as the goat.

(*To be continued.*)

## Figures and Tables

**Figure f1:**